# High Health-Related Quality of Life During Dendritic Cell Vaccination Therapy in Patients With Castration-Resistant Prostate Cancer

**DOI:** 10.3389/fonc.2020.536700

**Published:** 2020-10-26

**Authors:** Harm Westdorp, Jeroen H. A. Creemers, Inge M. van Oort, Niven Mehra, Simone M. Hins-de Bree, Carl G. Figdor, J. Alfred Witjes, Gerty Schreibelt, I. Jolanda M. de Vries, Winald R. Gerritsen, Petronella B. Ottevanger

**Affiliations:** ^1^ Department of Tumor Immunology, Radboud Institute for Molecular Life Sciences, Radboudumc, Nijmegen, Netherlands; ^2^ Department of Medical Oncology, Radboudumc, Nijmegen, Netherlands; ^3^ Oncode Institute, Nijmegen, Netherlands; ^4^ Department of Urology, Radboudumc, Nijmegen, Netherlands

**Keywords:** health-related quality of life (HRQoL), patient-reported outcomes (PROs), dendritic cell vaccination, immunotherapy, castration-resistant prostate cancer

## Abstract

**Background:**

Maintaining health-related quality of life (HRQoL) is highly desirable during systemic therapies for patients with castration-resistant prostate cancer (CRPC). Patient-reported outcome measures (PROs) were studied in our phase IIa trial on cellular-based immunotherapy with dendritic cells (DC).

**Methods:**

We treated 21 chemo-naive asymptomatic or minimally symptomatic patients with CRPC with maximally three cycles of DC vaccinations (ClinicalTrials.gov, NCT02692976). Here, we report the impact of DC vaccination on HRQoL. PROs were assessed using the EORTC-QLQ-C30, the EORTC-QLQ-PR25, Checklist Individual Strength (CIS20-R), and Beck Depression Inventory Primary Care questionnaires. Short-term and long-term vaccine-related effects on HRQoL were studied.

**Results:**

Questionnaires were collected at baseline (n=20), week 6 (n=19), week 12 (n=18), week 24 (n=13), week 50 (n=8) and week 100 (n=2). No clinically relevant differences in symptom-related outcome, functioning-related outcome, and Global Health Status were observed directly after the first cycle of DC vaccinations (week 6) and at follow-up (week 12) compared to baseline. HRQoL remained high throughout the vaccination cycle and six weeks afterward. In radiographic non-progressive patients, who continued DC vaccination, high HRQoL scores were observed up to one and two years after study enrolment.

**Conclusions:**

Patients with asymptomatic or minimally symptomatic CRPC show high HRQoL throughout DC-based immunotherapy. This is a clinically relevant finding in this older-aged patient population with advanced prostate cancer.

## Introduction

Prostate cancer (PCa) is the second leading cause of cancer-related death in men (1). Treatment of metastatic PCa is based on androgen deprivation therapy (ADT), which lowers circulating testosterone to castrate levels and inhibits tumor growth. Although primary ADT is initially very effective, in time, cancer cells become resistant. This stage is known as castration-resistant prostate cancer (CRPC) (2), a heterogeneous disease stage of advanced PCa. Since the approval of docetaxel in 2004, essential advances have been made in the treatment of metastatic CRPC (mCRPC). Over the past decade, landmark trials have shown an overall survival benefit in mCRPC for abiraterone, enzalutamide, cabazitaxel, radium-223, and sipuleucel-T, a dendritic cell (DC)-based immunotherapy (3–10). However, the optimal sequence of agents in mCRPC is unknown. The side effect profile is one of the parameters to help customize mCRPC treatment. Cancer-related symptom control is, therefore, a highly desirable health-related outcome, and patient-reported outcome measurements (PROMs) are included in most of the prospective phase II and III trials over the last five years (11–15).

In our randomized phase IIa study, we enrolled 21 asymptomatic or minimally symptomatic patients with chemotherapy-naive CRPC and treated them with myeloid DC (mDC), plasmacytoid DC (pDC) or combined myeloid and plasmacytoid DC vaccinations (ClinicalTrials.gov, NCT02692976). DCs can acquire and process antigen for subsequent presentation to T cells and thereby activate both naïve and memory immune cells (16). Immune responses, clinical responses, and the impact of DC vaccination on patient-reported outcomes (PROs) were studied. Earlier work on HRQoL in the context of adjuvant DC vaccination in patients with stage III melanoma showed HRQoL improvement was not hampered after surgery (17).

In the current trial in patients with asymptomatic or minimally symptomatic CRPC, we hypothesized that PROs would remain stable during in our DC subset vaccination trial.

## Patients and Methods

### Study Design and Patients

We studied PROMs during the open-label, randomized, phase IIa study of DC subset vaccination in 21 asymptomatic or minimally symptomatic patients with chemotherapy-naive CRPC. Patients were randomized (1:1:1) to mDC vaccinations, pDC vaccinations or combined mDC and pDC (combiDC) vaccinations. First, patients underwent a mononuclear cell apheresis. DC subsets were isolated from the apheresis material with magnetic beads. Subsequently, DCs were matured and loaded with tumor-associated antigens. DCs were injected under ultrasound guidance by an experienced radiologist or nuclear medicine specialist in a clinically benign lymph node. A cycle of DC vaccinations consisted of three biweekly intranodal injections. In the absence of radiographic disease progression, patients were eligible for two maintenance cycles of three biweekly vaccinations, with a six-month interval between cycles. PRO assessments were completed by patients before apheresis (baseline) and at weeks 6, 12, 24, 50, and 100 (**Supplementary Figure 1**). Adverse events were defined following the Common Terminology Criteria for Adverse Events version 4.0. The full study design, vaccine characteristics, eligibility criteria, immunological outcome parameters, clinical outcome parameters, and methodological details of the trial have been described elsewhere (18). The Dutch Central Committee on Research involving Human Subjects has approved the study (NL49143.000.14), and written informed consent was obtained from all patients. The ClinicalTrials.gov identifier of the study is NCT02692976. The study was conducted in accordance with the Good Clinical Practice guidelines and with the provisions of the Declaration of Helsinki (October 9^th^,2004).

### Patient-Reported Outcome Measures (PROMs)

The following PROMs were collected prospectively with four validated self-reported questionnaires: 1) the European Organisation for Research and Treatment of Cancer Quality of Life core Questionnaire C30 (EORTC-QLQ-C30) version 3.0, 2) the EORTC-QLQ-PR25, 3) the Checklist Individual Strength (CIS20-R) and 4) Beck Depression Inventory Primary Care (BDI-PC) (**Table 1**).

**Table 1:** Patient-reported outcome measurements.

Questionnaires	Subscales	Items (no.)	Possible range	Clinically relevant difference
**EORTC-QLQ-C30**	Functional	15	0–100*	Δ <10: non-significant Δ >10: moderate Δ >20: very much
	Symptoms	7	0–100*	
	Single-items	6^^^	0–100*	
	GHS	2	0–100*	
**EORTC-QLQ-PR25**				
**Functional** **Symptom**	Sexual active	2	0–100*	Δ <10: non-significant Δ >10: moderate Δ >20: very much
	Sexual functioning	4	0–100*	
	Urinary symptoms	8	0–100*	
	Bowel symptoms	4	0–100*	
	Hormonal treatment-related	6	0–100*	
	Incontinence aid	1	0–100*	
**CIS20-R**	CIS total	20	20–140	Score 27-35 (increased risk for fatigue)^$^ Score > 35 (severe fatigue)^$^
	CIS1 (subjective fatigue severity)	8	8–56	
	CIS2 (concentration)	5	5–35	
	CIS3 (motivation)	4	4–28	
	CIS4 (activity)	3	3–21	
**BDI-PC**	Depression inventory score	7	0–21	Score ≥4 (indicative of clinical depression)

BDI-PC, Beck Depression Inventory Primary Care; CIS, Checklist Individual Strength; EORTC, European Organisation for Research and Treatment of Cancer; GHS, Global Health Status.

^^^Incorporated single-item questions are about: 1) dyspnea; 2) insomnia; 3) appetite loss; 4) constipation; 5) diarrhea and 6) financial difficulties. ^*^Scores were linearly transformed to a 0 to 100 scale. ^$^Applicable for CIS1 only.

The EORTC-QLQ-C30 has five functional scales (physical, role, emotional, cognitive, and social functioning), three symptom scales (fatigue, nausea/vomiting, and pain), a Global Health Status (GHS) score and six single-item scores (dyspnea, insomnia, appetite loss, constipation, diarrhea, and financial difficulties). Patients rated the extent to which each statement was true for the previous week. Responses in the functional scales, symptom scales, and single-item scores included: “Not at all”, “A bit”, “Quite a bit”, and “Very much”. GHS scores range from “Very poor” to “Excellent”. Described scale scores, single-item scores, and GHS scores were linearly transformed to a 0 to 100 scale. A higher score on the functional scale, for GHS, and high symptom or single-item score represents a higher level of functioning, a high HRQoL, or a high symptomatology level, respectively. A clinically relevant difference was defined by a mean change of at least 10 points on a scale score (**Table 1**) (19, 20).

The EORTC-QLQ-PR25 is a PCa-specific validated questionnaire evaluating urinary and bowel symptoms, sexual activity and functioning, and ADT-induced side effects (hormonal treatment-related symptoms) (21). All QLQ-PR25 scores are linearly transformed to a scale from 0 to 100. QoL scores range from 0 to 100. A higher score on functioning-related domains is indicative of better functioning, where a higher symptom-related domain score is indicative of more symptomatology. Sexual functioning questions required reversing the response categories for 3 of 4 questions (question number 23–25). In line with the EORTC-QLQ-C30, a clinically relevant difference was defined by a mean change of at least 10 points on one of the scales.

The CIS20-R is a self-report questionnaire assessing 20 items encompassing four fatigue dimensions [subjective experience of fatigue (CIS1), reduction in concentration (CIS2), reduction in motivation (CIS3), and reduction in activity (CIS4)]. Patients rated the extent to which each statement was true for the previous two weeks on a 7-category scale (ranging from score 1 “Yes, that is true” to 7 “No, that is not true”). A CIS1 score of 35 or higher indicates severe fatigue. A score between 27 and 35 represents an increased risk for fatigue (22).

The BDI-PC questionnaire is one of the rating scales for identifying a mood disorder in medical outpatients. BDI-PC is a seven-item questionnaire with scores ranging from 0 to 21. Scores of 4 or higher are suggestive for a clinically relevant depression (23, 24).

### Statistical Analysis

A pre-planned descriptive comparison of PROMs was performed for this study. Outcome scores at group-level are tabulated and represented as mean with standard deviation. In order to compare group-level baseline scores with scores after either 6 or 12 weeks, the mean of the differences and 95% confidence interval are reported. Missing data were assumed to be absent at random for all analyses. Missing items from multi-item scales of the questionnaires were mean-imputed if at least half of the items from a scale were completed, according to the EORTC QLQ-C30 scoring guidelines (25). Descriptive statistics at a patient-level were graphically represented in heatmaps, generated using the R software environment for statistical computing and graphics (version 3.4.3).

## Results

### Patient Features

**Figure 1:**
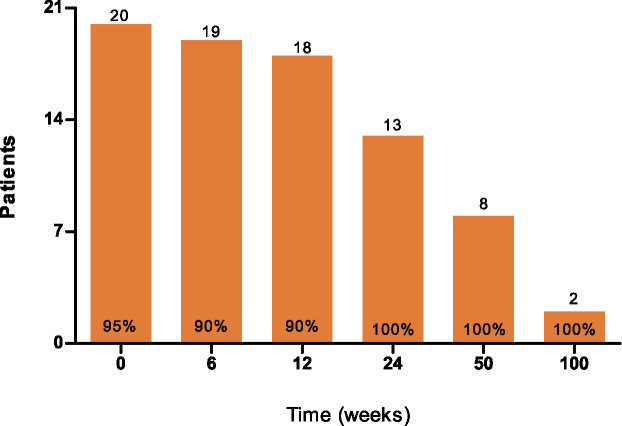
Completed patient-reported outcome measurements during the study period.

In total, 21 eligible patients with CRPC were included in his prospective study (**Supplementary Table 1** and **Supplementary Figure 1**) (18). At baseline, the median age was 67 years (range 53–82). Participants were treated with DC subset vaccines during the period from November 2015 until May 2018. All 21 patients (n=7 per arm) received at least one cycle of three biweekly blood-derived DC vaccinations and a skin test for immunomonitoring purposes. Thirteen patients also received a second cycle and seven patients a third vaccination cycle. 20 patients completed PROMs at baseline (95%), 19 patients at week 6 (90%), 18 patients at week 12 (90%), 13 patients at week 24 (100%), 8 patients at week 50 (100%) and 2 patients at week 100 (100%),which was dependent on patients remaining on study (**Figure 1**). One patient was excluded from the analyses for not filling out the PRO questionnaires at baseline.

### Clinical Vaccination-Related Adverse Events

Blood-derived DC vaccinations were well tolerated. No vaccine-related toxicity grade ≥3 was seen. In all vaccinated patients, at least one low-grade adverse event was reported. Flu-like symptoms were seen in 10 patients. In 8 of these 10 patients, fatigue lasted at least 1 day longer than the other flu-like symptoms or was present without other flu-like symptoms. Only 2 of these 10 patients had grade 1 or 2 fever. Four patients (3 treated with pDC vaccinations and 1 treated with combiDC vaccinations) were diagnosed with a grade 2 upper respiratory infection during the vaccination period. Three patients experienced grade 1 dizziness. In 2 patients, an injection site reaction developed upon the intranodal administration of DCs. Three patients had a hematoma post-vaccination. Two patients had a grade 1 headache after DC vaccination (**Supplementary Table 2**). More details and other laboratory side effects are described elsewhere (18).

### Symptom-Related Outcomes

**Figure 2:**
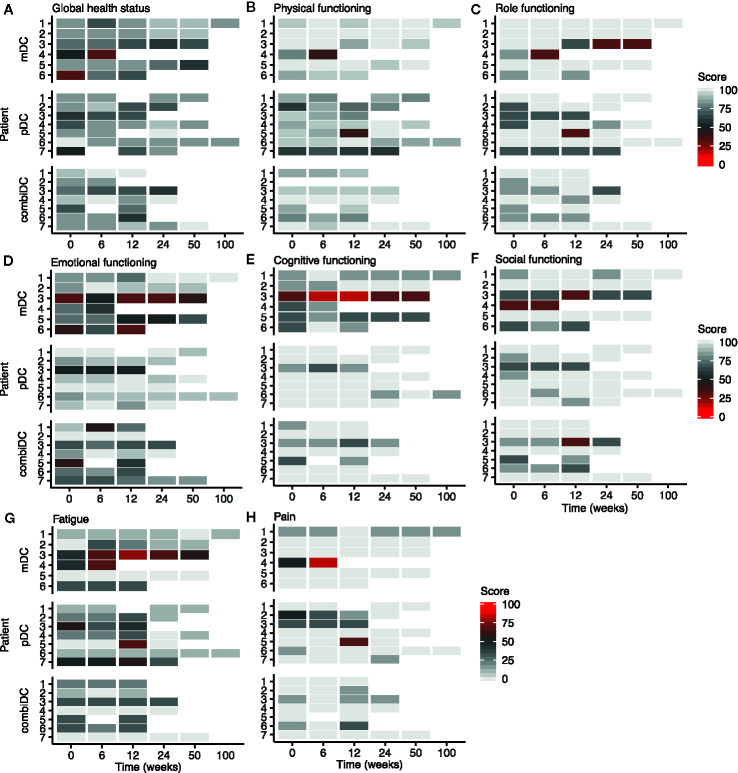
Heat map of functional and symptom scales of the EORTC-QLQ-C30 at different timepoints in patients with CRPC. **(A–F)** represent the functioning-related domains, **(G, H)** show two of the symptom-related domains. Other symptom scales are shown in **Supplementary Figure 2**.

**Figure 3:**
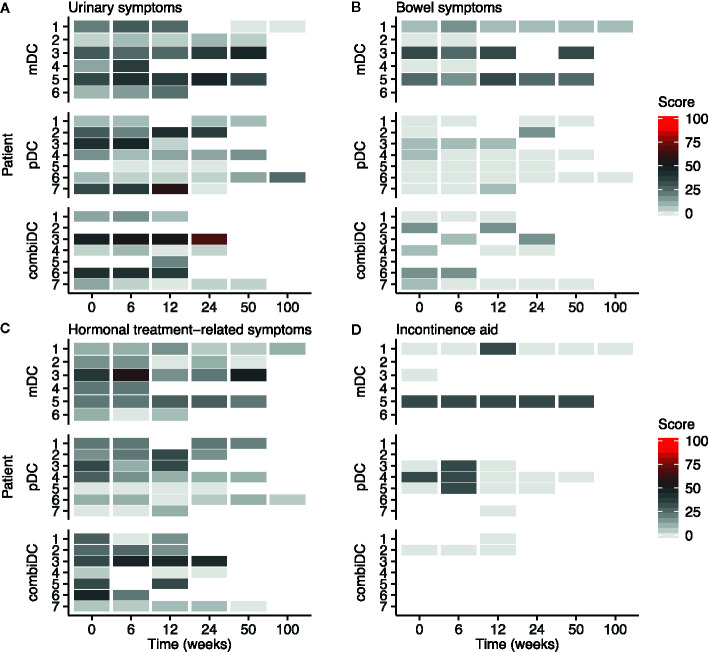
Heat map of symptom scales of the EORTC-QLQ-PR25 at different timepoints in patients with CRPC. **(A)** Urinary symptoms, **(B)** Bowel symptoms, **(C)** Hormonal treatment-related symptoms, **(D)** Incontinence aid. Functional scales are presented in **Supplementary Figure 3**.

**Figure 4:**
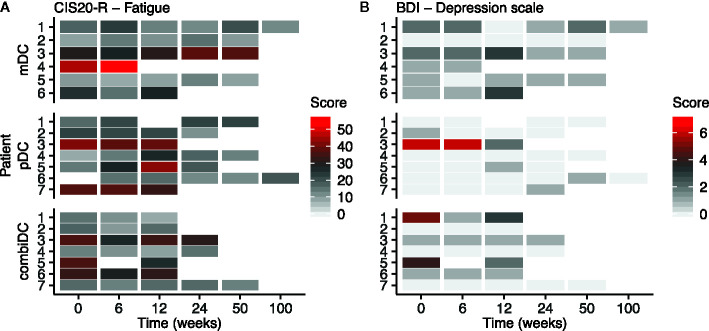
Heat map of CIS1 (subjective fatigue severity) and BDI-PC scores at different timepoints in patients with CRPC. **(A)** represents the CIS1 scores, **(B)** represents the BDI-PC scores. CIS2-4 scores are presented in **Supplementary Figure 4**.

Symptom-related domains of EORTC-QLQ-C30, EORTC-QLQ-PR25, and CIS1 of CIS20-R showed, at group-level, equal scores at baseline compared to week 6 and 12 (**Table 2**, **Figures 2**–**4** and **Supplementary Figure 2**). Most patients rated low symptom scores at baseline and during long-term follow-up (weeks 24, 50, and 100). At group-level, DC vaccination did not affect a patient’s level of symptoms.

**Table 2:** Patient-reported outcomes after the first dendritic cell vaccination cycle (week 6) and at follow-up (week 12).

	Score (SD)			Mean difference to baseline (CI)	
	Baseline	Week 6	Week 12	Week 6	Week 12
QLQ-C30 functional scales^*^					
Physical functioning	88.4 (11.9)	88.1 (14.7)	87.0 (16.4)	−0.4 (−6.6–5.9)	−2.1 (−9.7–5.6)
Role functioning	88.3 (13.4)	91.2 (17.9)	88.0 (18.8)	2.6 (−5.5–10.8)	0.0 (−11.4–11.4)
Emotional functioning	75.8 (20.2)	80.4 (19.4)	74.1 (21.9)	2.8 (−4.6–10.1)	−0.5 (−5.7–4.8)
Cognitive functioning	85.8 (18.2)	90.4 (20.3)	88.0 (21.2)	3.5 (−2.2–9.2)	1.9 (−3.0–6.7)
Social functioning	85.8 (18.2)	89.5 (17.8)	85.2 (22.8)	2.6 (−1.4–6.7)	−2.8 (−11.4–5.9)
GHS	75.4 (16.6)	79.2 (14.9)	76.6 (11.5)	1.9 (−4.9–8.6)	1.0 (−6.9–9.0)
QLQ-C30 symptom scales^*^					
Fatigue	21.9 (18.2)	23.1 (21.1)	29.0 (22.3)	1.8 (−4.2–7.7)	7.7 (−1.1–16.6)
Nausea/vomiting	0.0 (0.0)	0.0 (0.0)	1.9 (5.4)	0.0 (na)	1.9 (−0.8–4.5)
Pain	10.0 (16.6)	8.8 (21.1)	10.2 (18.2)	−1.8 (−7.0–3.5)	1.9 (−8.0–11.7)
Dyspnoea	11.7 (19.6)	12.3 (16.5)	14.8 (23.5)	0.0 (−9.3–9.3)	7.4 (−7.2–22.0)
Insomnia	25.0 (26.2)	25.9 (31.4)	22.2 (22.9)	0.0 (−8.0–8.0)	−1.9 (−8.8–5.0)
Loss of appetite	1.7 (7.5)	5.6 (12.8)	5.6 (12.8)	3.7 (−1.7–9.1)	3.7 (−1.7–9.1)
Constipation	12.2 (16.5)	7.0 (14.0)	5.6 (12.8)	−5.6 (−14.1–3.0)	−7.8 (−17.5–1.8)
Diarrhoea	3.3 (10.3)	0.0 (0.0)	3.7 (10.8)	−3.5 (−8.6–1.6)	0.0 (−8.0–8.0)
Financial difficulties	5.0 (22.4)	7.0 (17.8)	7.4 (18.3)	1.8 (−9.6–13.1)	1.9 (−10.2–13.9)
QLQ-PR25 functional scales^*^					
Sexual activity	75.4 (32.6)	88.9 (19.0)	84.3 (23.2)	13.0^#^ (−2.8–28.7)	10.8^#^ (−3.4–25.0)
Sexual functioning	56.3 (10.5)	58.3 (6.8)	50.0 (6.8)	−2.8 (−14.7–9.2)	−4 (−57.1–48.8)
QLQ-PR25 symptom scales^*^					
Urinary symptoms	21.5 (14.3)	22.6 (16.9)	20.9 (19.1)	2.4 (−1.9–6.7)	−0.5 (−8.3–7.2)
Bowel symptoms	7.8 (10.0)	6.1 (8.6)	9.0 (12.5)	−1.8 (−4.6–1.0)	−0.7 (−4.2–2.8)
Hormonal treatment-related	20.6 (13.0)	17.3 (15.3)	16.3 (13.8)	−3.5 (−9.3–2.4)	−2.5 (−8.1–3.1)
Incontinence aid	9.5 (16.3)	22.2 (17.2)	8.3 (15.4)	11.1^#^ (−7.0–29.2)	0.0 (−22.1–22.1)
**CIS20-R**					
Subjective fatigue severity	23.3 (12.0)	21.9 (11.7)	22.9 (11.1)	−0.3 (−3.4–2.8)	0.9 (−3.9–5.8)
Concentration	11.6 (7.1)	11.5 (7.1)	13.4 (7.9)	0.5 (−1.6–2.6)	1.9 (0.0–3.9)
Motivation	10.8 (5.6)	12.0 (6.1)	10.9 (5.1)	1.8 (−0.8–4.4)	1.4 (−0.5–3.4)
Physical activity	8.4 (4.9)	8.7 (5.2)	9.2 (4.7)	0.6 (−0.9–2.2)	1.5 (−0.3–3.3)
**BDI-PC**					
Depression inventory score	1.3 (1.8)	0.8 (1.4)	0.9 (1.2)	−0.3 (−0.7–0.1)	−0.4 (−1.0–0.3)

HRQoL scores range from 0 to 100. A higher score on functioning-related domains is indicative of better functioning, where a higher symptom-related domain score is

indicative of more symptomatology. ^#^Clinical relevant difference compared to baseline. This was defined by a mean difference of at least 10 points on the scale. BDI-PC, Beck Depression Inventory Primary Care; CIS, Checklist Individual Strength; EORTC, European Organisation for Research and Treatment of Cancer; GHS, Global Health Status; na, not applicable; SD, standard deviation. *Scores were linearly transformed to a 0 to 100 scale.

On average, an increase in symptoms was observed on the incontinence aid scale after 6 weeks (**Table 2**). At patient-level, this increase is represented by a temporary increase in symptoms of 2 patients (pDC-03 and pDC-05; **Figure 3D**). After 12 weeks, group-level values returned to baseline (**Table 2**).

At baseline, two patients (mDC-03 and combiDC-06) had a CIS1 score of 27–35, indicative of increased fatigue risk. At week 6 four patients (mDC-03, pDC-05, combiDC-03, and combiDC-06) and at week 12 six patients (mDC-03, mDC-06, pDC-07, combiDC-03, combiDC-05, and combiDC-06) showed an increased fatigue risk score, respectively.

Five patients scored CIS1 >35 at baseline, pinpointing at severe fatigue (mDC-04, pDC-03, pDC-07, combiDC-03, and combiDC-05). Severe fatigue scores were seen at week 6 in three patients (mDC-04, pDC-03, and pDC-07) and at week 12 in two pDC-treated patients (pDC-03 and pDC-05; **Figure 4A**).

### Functioning-Related Outcomes

Functioning-related domains of EORTC-QLQ-C30 and EORTC-QLQ-PR25 (physical functioning, role functioning, emotional functioning, cognitive functioning, social functioning, and sexual functioning) were not different throughout the study for all patients (**Table 2**, **Figures 2B–F** and **Supplementary Figure 3**). Using QLQ-C30, on average high physical, role, emotional, cognitive, and social functioning scores were observed throughout the trial (**Figures 2B–F**). For all patients, CIS2 (concentration), CIS3 (motivation), and physical activity (CIS4) showed no clinically significant difference, when comparing baseline to week 6 and week 12 (**Table 2** and **Supplementary Figure 4**).

The BDI-PC depression inventory score was similar at baseline compared to week 6 and 12 (**Table 2** and **Figure 4B**). Three patients (pDC-03, combiDC-01, and combiDC-05) had a baseline score of ≥4, indicative of clinical depression. Patient combiDC-05 started with antidepressive medication before apheresis. At week 6, one patient (pDC-03) had a score indicative of clinical depression. No patient scored ≥4 at week 12.

Sexual activity and functioning showed, however, dramatically low scores (**Supplementary Figure 3**). Sexual functioning scores were based on conditional questions in the PR25. Therefore, comparable scores were only obtained from up to 3 patients. Corresponding low scores were observed (**Supplementary Figure 3**). Low sexual activity and functioning scores were most certainly due to ongoing castration therapy.

At group-level, moderate improvement of sexual activity, indicated by an increase in score of ≥10, was found directly after the first vaccination cycle. This finding seemed to be consistent at week 12 (**Table 2**).

### Global Health Status

Symptom- and functioning-related domains represented a satisfactory wellbeing status for most patients. A high GHS reflected these results throughout the trial. GHS at baseline, week 6, and 12 were respectively 75, 79, and 77 (**Figure 2A**).

## Discussion

In this paper, the results of prospectively collected PRO questionnaires are described in patients with early CRPC treated with blood-derived DC vaccinations. To our knowledge, this is the first report of PROMs in advanced PCa patients treated with DC subsets. The questionnaires were well accepted by the patients, which yielded compliance rates of 90% or higher at every analyzed time point. DC vaccination was well tolerated, and the overall toxicity profile was acceptable with only grade 1-2 vaccine-related adverse events. Despite the limited study size, our results support the hypothesis that patients’ HRQoL would not deteriorate during treatment with DC vaccinations. In radiographic non-progressive patients, eligible for a second and third vaccination cycle, HRQoL remained stable up to one and two years after randomization, respectively. Given the low number of patients, these results should be interpreted with caution.

There are remarkable differences in fatigue outcomes between the questionnaires. Both the EORTC-QLQ-C30 and the CIS1 scored signs of fatigue. In the QLQ-C30 mean fatigue scores were 22, 23, and 29 at baseline, week 6 and 12, respectively, indicating relatively low mean fatigue scores (**Table 2**). This trend is also observed at patient-level (**Figure 2G**). However, the CIS1 outcome showed an increased fatigue risk score (27–35 points) or a severe fatigue score (>35 points) in approximately one-third of the patients at baseline, week 6 and 12 (**Figure 4A**). These results are relatively different from the QLQ-C30 outcome. This might be due to the setting in which both questionnaires were validated since QLQ-C30 is validated in cancer patients and CIS1 in chronic fatigue syndrome (22).

We report stable and often high HRQoL for patients with CRPC during treatment with DC vaccination. In comparison to landmark trials in minimally symptomatic mCRPC patients treated with radium-223, abiraterone, and enzalutamide, no improvement in HRQoL was seen during DC vaccination (11, 13–15). These findings need to be interpreted with caution: differences between the selected questionnaires hamper a clear and direct comparison, and might even stress the need for standardized HRQoL measurement in clinical trials in advanced PCa. However, a hypothesis-generating explorative comparison leads to some remarkable observations. Of interest in a minimally symptomatic chemo-naïve CRPC population is the treatment with second-generation anti-hormonal agents. While the PROSPER trial (enzalutamide versus placebo in non-metastatic CRPC) showed stable mean EORTC QLQ-PR25 scores throughout the trial (14), it reports the least squares mean difference from baseline for the hormonal treatment-related symptom score favoring the placebo group, suggesting an influence of side effects on HRQoL in these patients. Similar findings might be present in the results from the PREVAIL trial (enzalutamide versus placebo in asymptomatic or minimally symptomatic chemo-naive mCRPC); FACT-P scores (subscale “functional wellbeing”) declined over time in both arms (13). The influence of disease progression, however, needs to be considered. We did not see a clinically meaningful worsening in perceived cognitive functioning in our small study compared with enzalutamide-treated patients (**Figure 2E**) (26). Compared to the patient population of the ALSYMCPA trial (9), our patient population consisted of asymptomatic or minimally symptomatic chemo-naive CRPC patients. Baseline differences in the patient population, treatment-related toxicity profile, and the lack of a control arm complicates a direct comparison with these placebo-controlled landmark trials (11, 13, 14). A control arm could correct for a possible placebo-effect on HRQoL caused by the advantages of taking part in a clinical trial, such as frequent PSA measurements and 3-monthly imaging.

A potential pitfall of a study without a control group to monitor PROs is the induction of selection bias. Questionnaire completion rates were not merely caused by compliance but were influenced by study continuation based on the absence of radiological progression (**Figure 1**). One and two patients did not complete the questionnaires at weeks 6 and 12, respectively (**Figures 1**–**4** and **Supplementary Figures 2–4**). The missing patient at week 6 had radiographic progressive disease. At week 12, missing data came from one progressive patient and one non-progressive patient. Also, the HRQoL outcome of DC-vaccinated radiographic non-progressive patients (all questionnaires completed ≥ week 24) could reflect the disease biology and not a direct effect of DC vaccinations itself. Therefore, these findings on HRQoL need to be interpreted cautiously, and confirmation is required.

Studied PROMs are a selection of the available validated questionnaires. The four used questionnaires were selected to ensure a full picture of the patient’s HRQoL and are based on expected symptoms of DC vaccinations. We focused on symptom-related- (EORTC-QLQ-C30, EORTC-QLQ-PR25, and CIS20-R), functional-related- (EORTC-QLQ-C30 and EORTC-QLQ-PR25) and emotional-related wellbeing (CIS20-R and BDI-PC). Examples of other questionnaires that could be valuable in patients with CRPC are the EuroQoL EQ-5D (validated for a general population) (27), the Functional Assessment of Cancer Therapy-Prostate (FACT-P; a PCa-specific questionnaire) (28) and the EORTC-QLQ-BM22 (for patients with bone metastases) (29).

In conclusion, we observed a high HRQoL in patients with CRPC that participated in our DC vaccination trial. Most patients started with a high HRQoL, which did not deteriorate throughout the trial. In radiographic non-progressive patients, who continued DC vaccination after one cycle, high HRQoL scores were observed one and two years after study enrolment. This is a clinically relevant finding for elderly patients with CRPC.

## Data Availability Statement

All datasets generated for this study are included in the article/**Supplementary Material**.

## Ethics Statement

The Dutch Central Committee on Research involving Human Subjects has approved the study (NL49143.000.14), and written informed consent was obtained from all patients. The study was conducted in accordance with the Good Clinical Practice guidelines and with the provisions of the Declaration of Helsinki (October 9th, 2004).

## Author Contributions

The study is conceptualized by CF, JW, IV, and WG, and designed by HW, CF, GS, IV, and WG. The data is collected by HW, JC, IO, and SH-B. The data analysis and interpretation are performed by HW, JC, GS, IV, WG, and PO. HW and JC performed the statistical analysis. The manuscript is prepared by HW and JC, and subsequently edited by HW, JC, IO, NM, GS, IV, WG, and PO. All authors contributed to the article and approved the submitted version.

## Funding

This work was supported by Stichting Afweer Tegen Kanker, Dr. Paul A.J. Speth Stichting and the Radboudumc. CF received a ERC Adv Grant ARTimmune (834618) and the NWO Spinoza grant. IV received a NWO-Vici grant (918.14.655) and the H2020 EU Grant PROCROP (Grant Number 635122).

## Conflict of Interest

IO has received grants, personal fees, and non-financial support from Astellas; grants and personal fees from Janssen-Cilag; grants and personal fees from Bayer and personal fees from Sanofi. NM reports personal fees from Bayer, grants and personal fees from Jansen-Cilag, personal fees from Merck Sharp & Dohme, grants and personal fees from Roche, grants and personal fees from Astellas, and grants and personal fees from Sanofi. JW reports personal fees from Sanofi, Merck Sharp & Dohme, Bristol‐Myers Squibb, and Roche. WG reports speaker fees from Merck Sharp & Dohme and ESMO; advisory board fees from Bayer, Bristol‐Myers Squibb, IMS Health/iQVia, Janssen‐Cilag, Merck Sharp & Dohme, and Sanofi; research grants from Astellas, Bayer, Janssen-Cilag, and Sanofi. PO received fees from advisory boards of Pfizer, Clovis, Tesaro, and Merck Sharp & Dohme.

The remaining authors declare that the research was conducted in the absence of any commercial or financial relationships that could be construed as a potential conflict of interest.

The handling Editor declared a past co-authorship with several of the authors GS, IV.

## Abbreviations

ADT, Androgen deprivation therapy; BDI-PC, Beck Depression Inventory Primary Care; CIS, Checklist Individual Strength; (m)CRPC, (metastatic) Castration-resistant prostate cancer; DC, Dendritic cells; GHS, Global health status; HRQoL, Health-related quality of life; mDC, myeloid dendritic cells; PCa, Prostate cancer; pDC, Plasmacytoid dendritic cells; PRO, Patient-reported outcome; PROM, Patient-reported outcome measure.

## References

[1] Siegel RL Miller KD Jemal A Cancer statistics, 2019. CA Cancer J Clin (2019) 69(1):7–34. 10.3322/caac.21551 30620402

[2] Westdorp H Skold AE Snijer BA Franik S Mulder SF Major PP Immunotherapy for prostate cancer: lessons from responses to tumor-associated antigens. Front Immunol (2014) 5:191. 10.3389/fimmu.2014.00191 24834066 PMC4018526

[3] de Bono JS Logothetis CJ Molina A Fizazi K North S Chu L Abiraterone and increased survival in metastatic prostate cancer. N Engl J Med (2011) 364(21):1995–2005. 10.1056/NEJMoa1014618 21612468 PMC3471149

[4] Scher HI Fizazi K Saad F Taplin ME Sternberg CN Miller K Increased survival with enzalutamide in prostate cancer after chemotherapy. New Engl J Med (2012) 367(13):1187–97. 10.1056/NEJMoa1207506 22894553

[5] Ryan CJ Smith MR Fizazi K Saad F Mulders PF Sternberg CN Abiraterone acetate plus prednisone versus placebo plus prednisone in chemotherapy-naive men with metastatic castration-resistant prostate cancer (COU-AA-302): final overall survival analysis of a randomised, double-blind, placebo-controlled phase 3 study. Lancet Oncol (2015) 16(2):152–60. 10.1016/S1470-2045(14)71205-7 25601341

[6] Beer TM Armstrong AJ Rathkopf DE Loriot Y Sternberg CN Higano CS Enzalutamide in metastatic prostate cancer before chemotherapy. New Engl J Med (2014) 371(5):424–33. 10.1056/NEJMoa1405095 PMC4418931 24881730

[7] de Bono JS Oudard S Ozguroglu M Hansen S Machiels JP Kocak I Prednisone plus cabazitaxel or mitoxantrone for metastatic castration-resistant prostate cancer progressing after docetaxel treatment: a randomised open-label trial. Lancet (2010) 376(9747):1147–54. 10.1016/S0140-6736(10)61389-X 20888992

[8] Eisenberger M Hardy-Bessard AC Kim CS Geczi L Ford D Mourey L Phase III Study Comparing a Reduced Dose of Cabazitaxel (20 mg/m(2)) and the Currently Approved Dose (25 mg/m(2)) in Postdocetaxel Patients With Metastatic Castration-Resistant Prostate Cancer-PROSELICA. J Clin Oncol (2017) 35(28):3198–206. 10.1200/JCO.2016.72.1076 28809610

[9] Parker C Nilsson S Heinrich D Helle SI O’Sullivan JM Fossa SD Alpha emitter radium-223 and survival in metastatic prostate cancer. New Engl J Med (2013) 369(3):213–23. 10.1056/NEJMoa1213755 23863050

[10] Kantoff PW Higano CS Shore ND Berger ER Small EJ Penson DF Sipuleucel-T immunotherapy for castration-resistant prostate cancer. New Engl J Med (2010) 363(5):411–22. 10.1056/NEJMoa1001294 20818862

[11] Nilsson S Cislo P Sartor O Vogelzang NJ Coleman RE O’Sullivan JM Patient-reported quality-of-life analysis of radium-223 dichloride from the phase III ALSYMPCA study. Ann Oncol (2016) 27(5):868–74. 10.1093/annonc/mdw065 PMC4843190 26912557

[12] Heidenreich A Chowdhury S Klotz L Siemens DR Villers A Ivanescu C Impact of Enzalutamide Compared with Bicalutamide on Quality of Life in Men with Metastatic Castration-resistant Prostate Cancer: Additional Analyses from the TERRAIN Randomised Clinical Trial. Eur Urol (2017) 71(4):534–42. 10.1016/j.eururo.2016.07.027 27497762

[13] Loriot Y Miller K Sternberg CN Fizazi K De Bono JS Chowdhury S Effect of enzalutamide on health-related quality of life, pain, and skeletal-related events in asymptomatic and minimally symptomatic, chemotherapy-naive patients with metastatic castration-resistant prostate cancer (PREVAIL): results from a randomised, phase 3 trial. Lancet Oncol (2015) 16(5):509–21. 10.1016/S1470-2045(15)70113-0 25888263

[14] Tombal B Saad F Penson D Hussain M Sternberg CN Morlock R Patient-reported outcomes following enzalutamide or placebo in men with non-metastatic, castration-resistant prostate cancer (PROSPER): a multicentre, randomised, double-blind, phase 3 trial. Lancet Oncol (2019) 20(4):556–69. 10.1016/S1470-2045(18)30898-2 30770294

[15] Khalaf DJ Sunderland K Eigl BJ Kollmannsberger CK Ivanov N Finch DL Health-related Quality of Life for Abiraterone Plus Prednisone Versus Enzalutamide in Patients with Metastatic Castration-resistant Prostate Cancer: Results from a Phase II Randomized Trial. Eur Urol (2019) 75(6):940–7. 10.1016/j.eururo.2018.12.015 30591354

[16] Steinman RM Decisions about dendritic cells: past, present, and future. Annu Rev Immunol (2012) 30:1–22. 10.1146/annurev-immunol-100311-102839 22136168

[17] Bloemendal M Rietveld MJA van Willigen WW Gerritsen WR Figdor CG Bonenkamp JJ Health-related quality of life analysis in stage III melanoma patients treated with adjuvant dendritic cell therapy. Clin Transl Oncol (2019) 21(6):774–80. 10.1007/s12094-018-1987-0 30465182

[18] Westdorp H Creemers JHA van Oort IM Schreibelt G Gorris MAJ Mehra N Blood-derived dendritic cell vaccinations induce immune responses that correlate with clinical outcome in patients with chemo-naive castration-resistant prostate cancer. J Immunother Cancer (2019) 7(1):302. 10.1186/s40425-019-0787-6 31727154 PMC6854814

[19] Osoba D Rodrigues G Myles J Zee B Pater J Interpreting the significance of changes in health-related quality-of-life scores. J Clin Oncol (1998) 16(1):139–44. 10.1200/JCO.1998.16.1.139 9440735

[20] King MT The interpretation of scores from the EORTC quality of life questionnaire QLQ-C30. Qual Life Res (1996) 5(6):555–67. 10.1007/Bf00439229 8993101

[21] van Andel G Bottomley A Fossa SD Efficace F Coens C Guerif S An international field study of the EORTC QLQ-PR25: a questionnaire for assessing the health-related quality of life of patients with prostate cancer. Eur J Cancer (2008) 44(16):2418–24. 10.1016/j.ejca.2008.07.030 18774706

[22] Vercoulen JH Swanink CM Fennis JF Galama JM van der Meer JW Bleijenberg G Dimensional assessment of chronic fatigue syndrome. J Psychosom Res (1994) 38(5):383–92. 10.1016/0022-3999(94)90099-X 7965927

[23] Beck AT Steer RA Ball R Ciervo CA Kabat M Use of the Beck Anxiety and Depression Inventories for Primary Care with Medical Outpatients. Assessment (1997) 4(3):211–9. 10.1177/107319119700400301 26613770

[24] Goedendorp MM Gielissen MF Verhagen CA Peters ME Bleijenberg G Severe fatigue and related factors in cancer patients before the initiation of treatment. Br J Cancer (2008) 99(9):1408–14. 10.1038/sj.bjc.6604739 PMC2579682 18941462

[25] Fayers P Aaronson NK Bjordal K Groenvold M Curran D Bottomley A EORTC QLQ-C30 Scoring Manual. 3rd edn European Organisation for Research and Treatment of Cancer (2001).

[26] Thiery-Vuillemin A Poulsen MH Lagneau E Ploussard G Birtle A Dourthe LM Impact of abiraterone acetate plus prednisone or enzalutamide on fatigue and cognition in patients with metastatic castration-resistant prostate cancer: initial results from the observational AQUARiUS study. ESMO Open (2018) 3(5):e000397. 10.1136/esmoopen-2018-000397 30116592 PMC6088345

[27] EuroQol G EuroQol–a new facility for the measurement of health-related quality of life. Health Policy (1990) 16(3):199–208. 10.1016/0168-8510(90)90421-9 10109801

[28] Cella D Nichol MB Eton D Nelson JB Mulani P Estimating clinically meaningful changes for the Functional Assessment of Cancer Therapy–Prostate: results from a clinical trial of patients with metastatic hormone-refractory prostate cancer. Value Health (2009) 12(1):124–9. 10.1111/j.1524-4733.2008.00409.x 18647260

[29] Chow E Nguyen J Zhang L Tseng LM Hou MF Fairchild A International field testing of the reliability and validity of the EORTC QLQ-BM22 module to assess health-related quality of life in patients with bone metastases. Cancer (2012) 118(5):1457–65. 10.1002/cncr.26410 21837676

