# Supplementary material for: High Health-Related Quality of Life During Dendritic Cell Vaccination Therapy in Patients With Castration-Resistant Prostate Cancer
Source: Front Oncol. 2020 Oct 26;10:536700. doi: 10.3389/fonc.2020.536700 (PMC7649342; doi:10.3389/fonc.2020.536700)
Supplement: Supplementary file 1 [file DataSheet_1.docx]

Supplementary Material

# Supplementary Figures and Tables

## Supplementary Figures


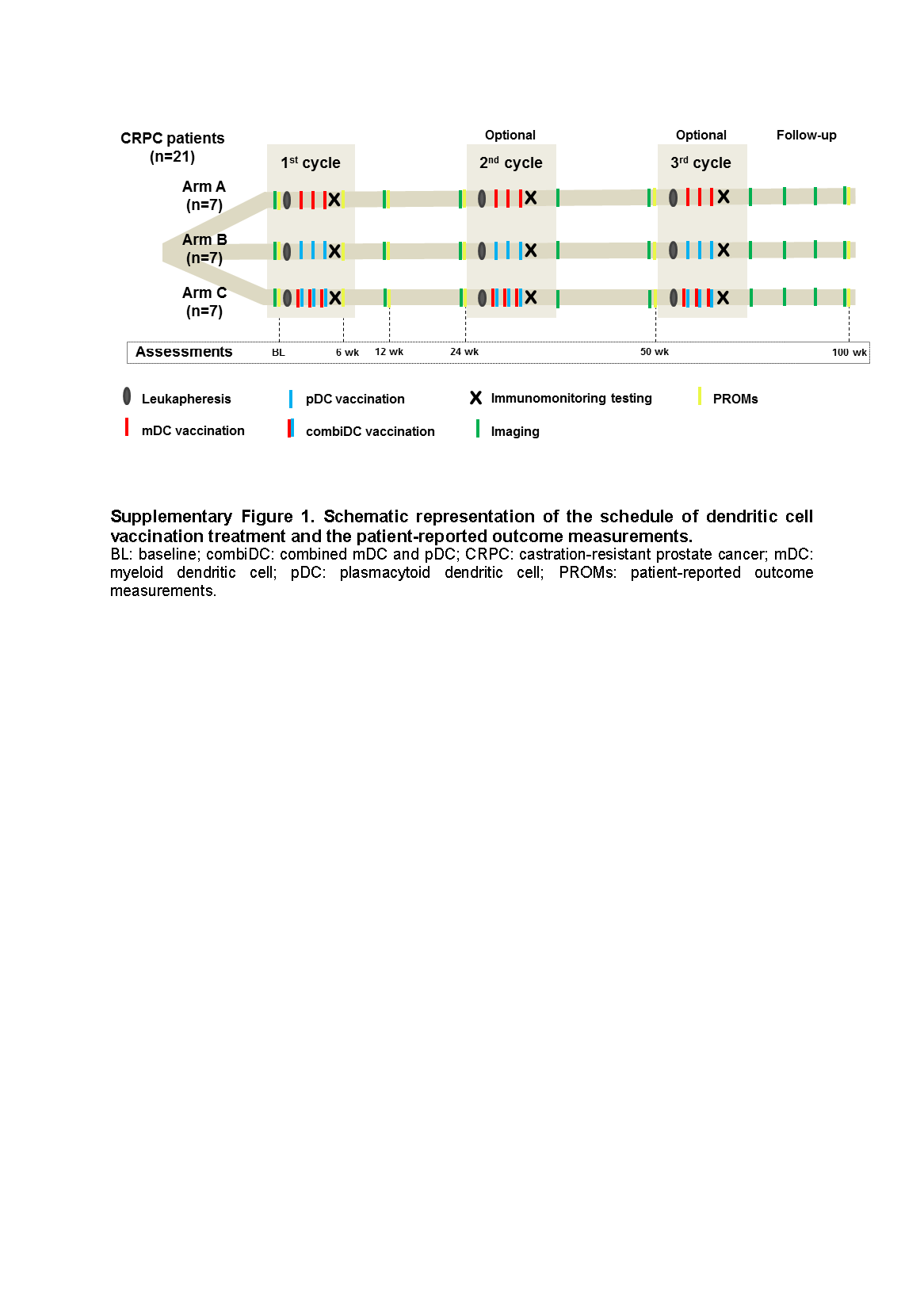


**Supplementary Figure 1.** Schematic representation of the schedule of dendritic cell vaccination treatment and the patient-reported outcome measurements. BL, baseline; combiDC, combined mDC and pDC; CRPC, castration-resistant prostate cancer; mDC, myeloid dendritic cell; pDC, plasmacytoid dendritic cell; PROMs, patient-reported outcome measurements.


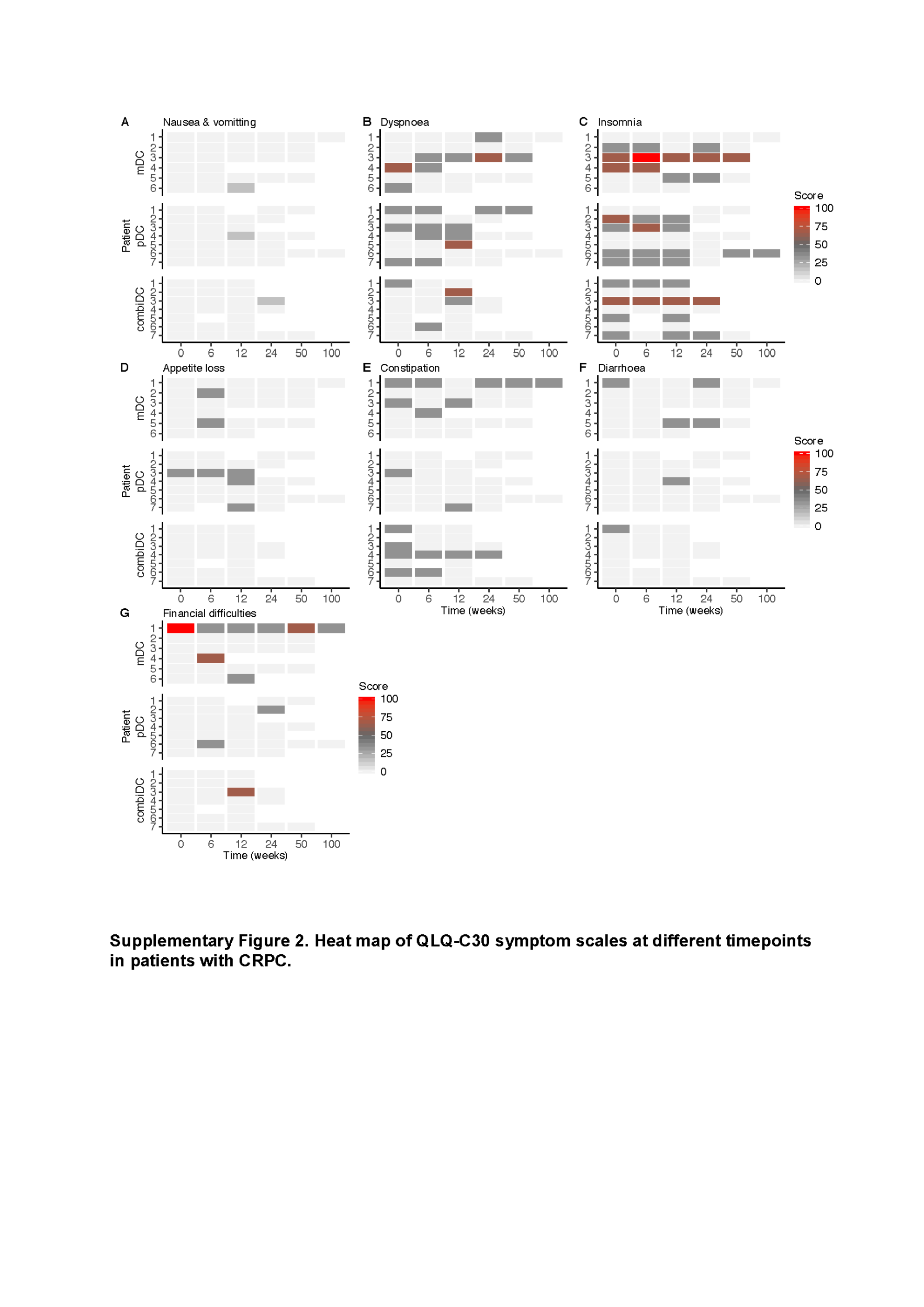


**Supplementary Figure 2.** Heatmap of QLQ-C30 symptom scales at different time points in patients with CRPC.


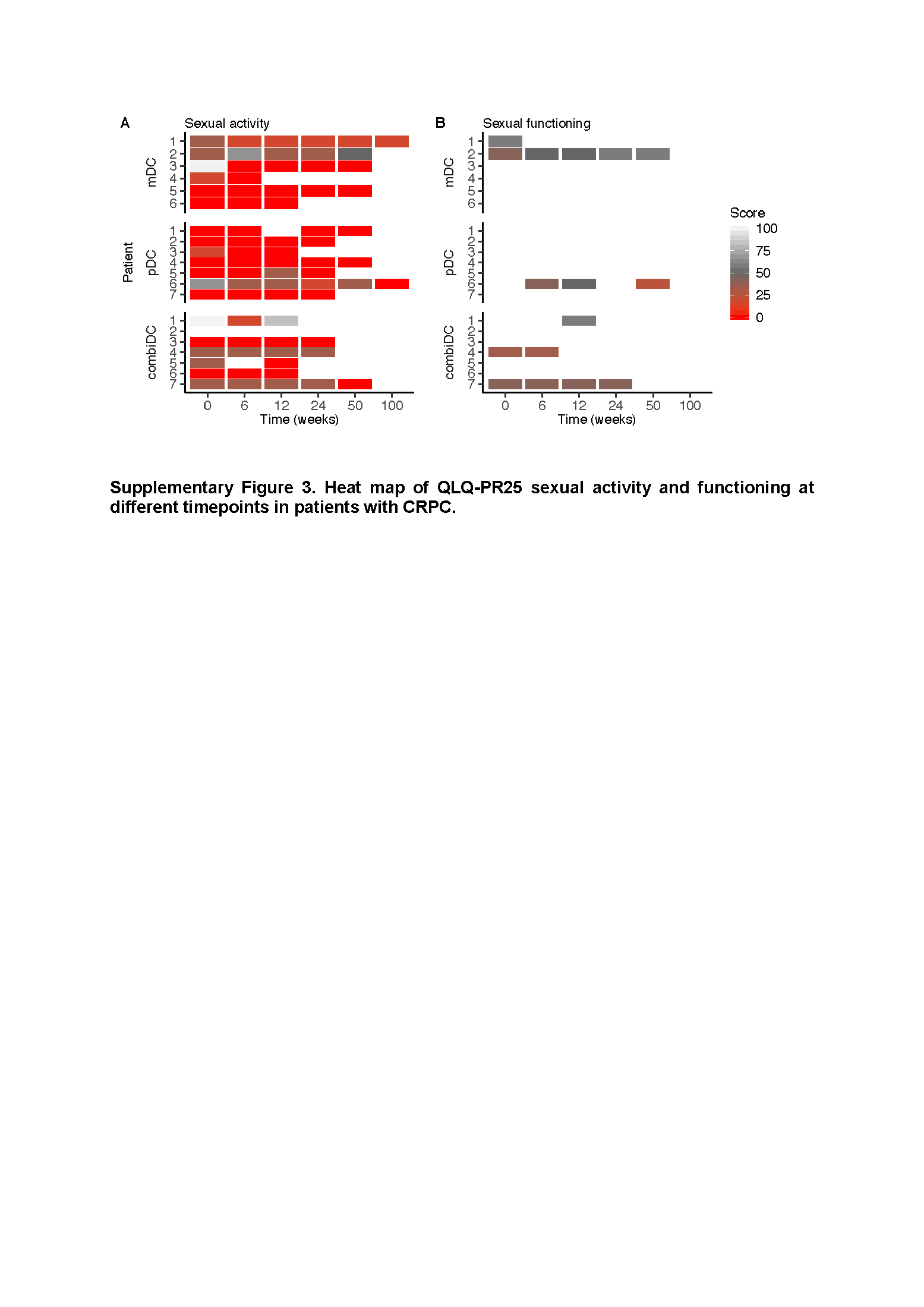


**Supplementary Figure 3.** Heatmap of QLQ-PR25 sexual activity and functioning at different time points in patients with CRPC.


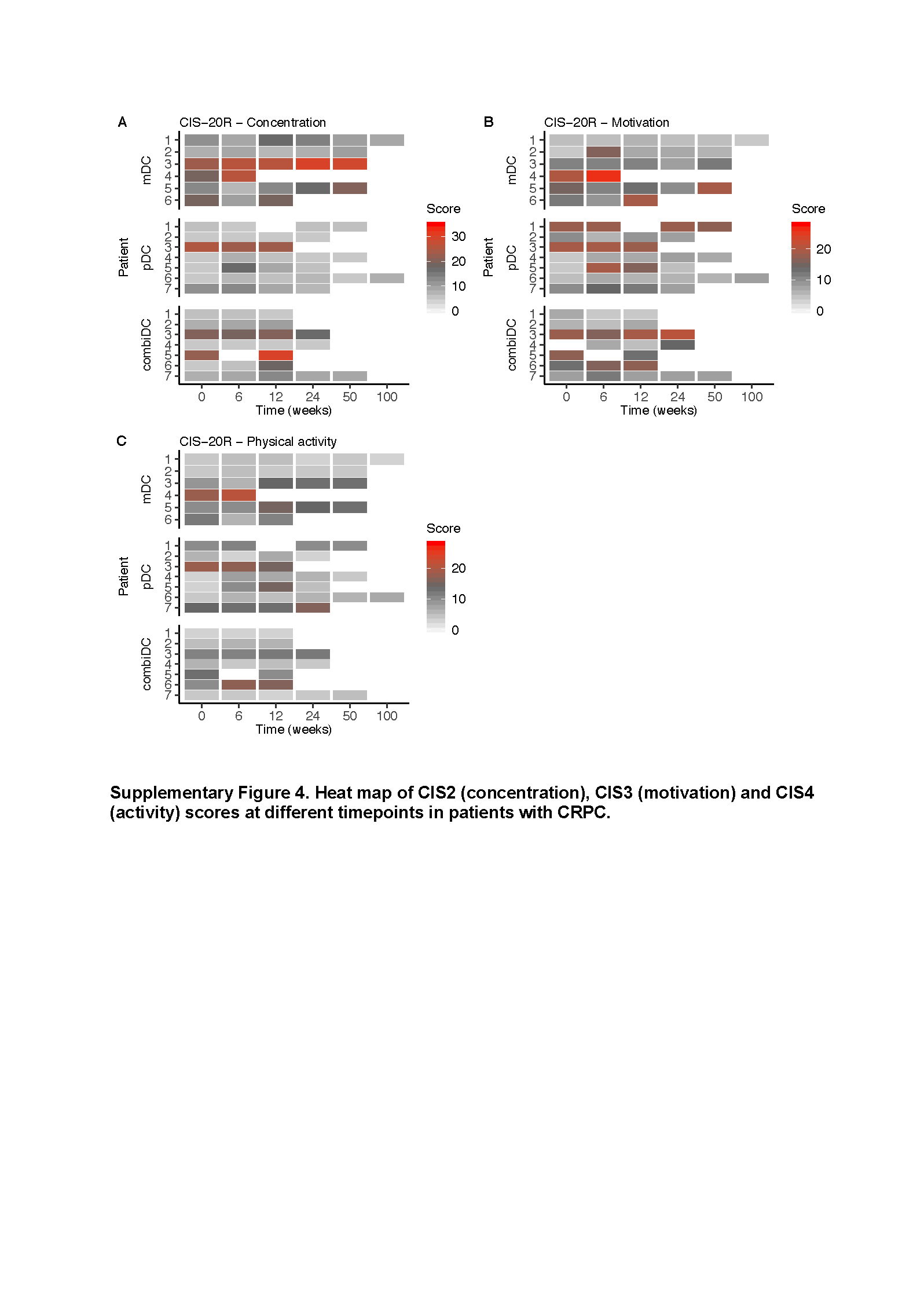


**Supplementary Figure 4**. Heatmap of CIS2 (concentration), CIS3 (motivation) and CIS4 (activity) scores at different time points in patients with CRPC.

## Supplementary Tables

**Supplementary Table 1.** Baseline clinical characteristics of enrolled patients with CRPC. Abbreviations: ALP, alkaline phosphatase; combiDC, combination of myeloid and plasmacytoid dendritic cell vaccination; CRPC, castration-resistant prostate cancer; DC, dendritic cells; LN, lymph node; LDH, lactate dehydrogenase; mDC, myeloid dendritic cell vaccination; pDC, plasmacytoid dendritic cell vaccination; PSA, prostate-specific antigen. ^*^ Measurable disease sites were determined on 68Ga-PSMA PET plus contrast-enhanced CT scans with a 3 mm slide thickness for Response Evaluation Criteria In Solid Tumors (RECIST) version 1.1 and ferumoxtran-10-enhanced MRIs and MRI bones and lymph nodes for Prostate Cancer Clinical Trials Working Group 2 (PCWG2) criteria. Measurable lesions were measured in at least one dimension with longest diameter ≥10 mm. Small lesions (longest diameter <10 mm or pathological lymph nodes with <15 mm short axis), are considered non-measurable disease. Bone metastases were documented and assessed according to PCWG2 criteria.

| DC vaccination-treated patient | Age (median; years (range)) | Gleason score (%) | Disease sites^*^  (%) | Baseline PSA  (median; ug/l (range)) | Baseline LDH  (%) | Baseline ALP  (%) |
| --- | --- | --- | --- | --- | --- | --- |
| combiDC (n=7) | 67 (53-74) | <8 (29)  8 (43)  >8 (29) | No measurable disease (14)  LN (0)  LN + bone (29)  Bone (43) | 18.0 (3.7-120.0) | < 200 U/l (71)  > 200 U/l (29) | < 1.0 ULN (86)  > 1.0 ULN (14) |
| pDC (n=7) | 70 (59-82) | <8 (29)  8 (0)  >8 (71) | No measurable disease (0)  LN (43)  LN + bone (29)  Bone (29) | 6.3 (2.6-19.0) | < 200 U/l (71)  > 200 U/l (29) | < 1.0 ULN (86)  > 1.0 ULN (14) |
| mDC (n=7) | 67 (60-78) | <8 (43)  8 (14)  >8 (43) | No measurable disease (29)  LN (14)  LN + bone (29)  Bone (29) | 10.0 (4.6-260.0) | < 200 U/l (57)  > 200 U/l (43) | < 1.0 ULN (86)  > 1.0 ULN (14) |

**Supplementary Table 2.** Vaccination-related adverse events. CTCAE 4.0: Common Terminology Criteria for Adverse Events version 4.0. ^*^ Attributed by study investigators. ^^^ Other includes: nausea (1), vomiting (1) and diarrhea (1).

|  | Patients (n=21) |  |
| --- | --- | --- |
| Reported toxicity (CTCAE 4.0)* | **Grade 1-2** | **Grade ≥3** |
| Any toxicity | **21** | **0** |
| Anemia | **15** | **0** |
| Flu-like symptoms | **10** | **0** |
| Fatigue | **8** | **0** |
| Upper respiratory infection | **4** | **0** |
| Dizziness | **3** | **0** |
| Hematoma | **3** | **0** |
| Injection site reaction | **2** | **0** |
| Fever | **2** | **0** |
| Headache | **2** | **0** |
| Other^^^ | **1** | **0** |
